# Field application of *Bacillus subtilis* and *Aureobasidium pullulans* to reduce *Monilinia laxa* post-harvest rot on cherry

**DOI:** 10.1007/s10658-022-02508-8

**Published:** 2022-04-26

**Authors:** Sophia Bellamy, Michael Shaw, Xiangming Xu

**Affiliations:** 1 Pest & Pathogen Ecology, NIAB, West Malling, KY ME19 6BJ UK; 2 School of Agriculture, Policy and Development, University of Reading, Reading, Berkshire UK

**Keywords:** Biocontrol, *Monilinia* spp., Brown rot, Stone fruit, Cherry, *Prunus avium*, Post-harvest rot

## Abstract

Brown rot caused by *Monilinia* spp. is one of the most important diseases in stone fruits worldwide. Latent infections of fruit by the pathogen often manifest once the fruit is ripe, leading to post-harvest rots. Two microbial strains (*Bacillus subtilis -*B91 and *Aureobasidium pullulans* - Y126) have shown antagonistic properties against *M. laxa* in previous studies. This study assessed the reduction in post-harvest rot of cherry by *M. laxa* when the two biocontrol (BCAs) strains were applied pre-harvest under field conditions to fruit artificially inoculated with *M. laxa*. The experiment was carried out for two consecutive years in cherry orchards in Kent. When applied pre-harvest, both strains (B91 and Y126) reduced the incidence of post-harvest rots by 30% - 60%. This promising result helps towards the commercialisation of the two strains, which would reduce reliance on fungicides in commercial cherry production.

**Supplementary Information:**

The online version contains supplementary material available at 10.1007/s10658-022-02508-8.

*Monilinia* spp., the causal agent of brown rot (Holb, 2019) not only causes blossom wilts and fruit rots in orchards, the pathogen can also cause latent infections of fruit, that lead to post-harvest rot. Latent infections are characterised by *M. laxa* penetrating immature fruits but remaining dormant (latent) until conditions are more favourable, often near or after harvest manifesting as post-harvest rot (Larena et al., 2021). In the first stage of fruit formation, the green fruitlet is photosynthetically active and is susceptible to infection (Oliveira Lino et al., 2016). This susceptibility is thought to be due to the active stomata forming an easy entry point for the fungi. The following stage, pit hardening, is the least susceptible due to the increase in secondary compounds including phenolics such as catechin and epicatechin that have antifungal properties. Once the pericarp forms and hardens, the concentration of phenolic compounds declines and that of sugars increases, leading to increased susceptibility to *M. laxa*. Physical changes such as thinning or fracturing of the cuticle associated with fruit maturity can also increase fruit susceptibility (Gatto et al., 2011; Oliveira Lino et al., 2016).

Latent infections that occurred in the field can quickly develop into visual rots post-harvest and easily spread via contact within cold storage(Garcia-Benitez et al., 2020). The ability of *M. laxa* to grow rapidly at 5–10 °C can lead to the development of fruit rot post-harvest. The control of the disease is still heavily reliant on the use of fungicides, particularly pre- post-harvest (Crowley-Gall et al., 2022) but the use of fungicides is increasingly restricted in commercial agriculture. This has led to an increase in research into alternative management methods, such as biological control (Usall et al., 2015). In 2013, two microbial strains (*Aureobasidium pullulans* - Y126, a yeast-like fungus; *Bacillus subtilis* - B91, a bacterium) were isolated from cherry within the UK (Rungjindamai et al., 2013). These two strains showed promise in suppressing *M. laxa* sporulation on mummified fruit as well as being able to survive over a range of temperatures under lab conditions.

Y126 and B91 are from two species (*Aureobasidium pullulans* and *B. subtilis*) that have strains already formulated into commercial biocontrol products; Serenade (Serenade Max®, Bayer CropScience) is a *B. subtilis* biopesticide using strain QST713 and is approved by the European Union. Bio-ferm has produced two formulated *Aureobasidium pullulans* biopesticides ‘BoniProtect’ and ‘Blossom Protect’ that are approved to manage *Botrytis cinerea* and *Penicillium expansum* (Bellamy et al., 2021). This gives promise that Y126 and B91 can also be formulated into biopesticides and approved for commercial agriculture.

Though many previous studies have focused on the post-harvest application of biocontrols, pre-harvest applications of microbial antagonists could also be an effective control measure to reduce post-harvest decay. A pre-harvest application would not only reduce pre-harvest infection and disease development but also allow beneficial microbes to colonise the fruit surface before harvest, and so reduce secondary contact spread in cold storage (Ippolito & Nigro, 2000). The present study was conducted in 2019 and 2020 to test the hypothesis that B91 and Y126 applied close to harvest would reduce post-harvest cherry fruit rot caused by *M. laxa*.

In 2019, both products, or distilled water, were applied 24 h before artificial inoculation of fruit with *M. laxa,* giving a total of three treatments: fruit treated with each of the two biocontrol microbes and fruit treated only with sterile distilled water as a control. In 2020, there were nine treatments: [1–8] four products – fungicide (a succinate dehydrogenase inhibitor-quinone outside inhibitor mix), sterile distilled water and the two biocontrol microbes (B91 and Y126)) applied either 24 h before or 24 h after *M. laxa* inoculation, and [9] fruits that were not subjected to artificial inoculation with *M. laxa* or biocontrol microbes but harvested to assess the background level of brown rot. The treatment applied before and after *M. laxa* inoculation was to assess preventative and curative protection efficacies of the two strains, respectively.

Single colonies of B91 and Y126 were grown in liquid media (liquid broth and potato dextrose broth, respectively) for 24 h on a shaking incubator (180 rpm, 25 °C). Propagule concentration was estimated with a spectrophotometer and adjusted to OD_600_ 0.2 (B91) and 0.01 (Y126) to achieve a propagule concentration of ~1 × 10^8^ CFU ml^−1^. The inoculum was applied to the fruit in orchards as a cell suspension with the culture medium, hence including inhibitory compounds, if any, in the medium. *M. laxa* inoculum was grown on ripe plums. Spores were harvested from the surface of the fruit using a sterile scalpel, then suspended in sterile distilled water and adjusted to 1 × 10^5^ spores ml^−1^ with a haemocytometer. The biocontrol suspensions and sterile distilled water were transferred to handheld sprayers. Fungicide Lunar sensation (250 g/L fluopyram and 250 g/L trifloxystrobin, Bayer, www.cropscience.bayer.co.uk) was prepared at 0.3 ml/L according to the manufacturer’s instructions.

In 2019, the experiment was carried out on three trees of cultivar Kordia in an open-air orchard at NIAB East Malling. Three similar branches, at the same height on each tree, were selected, one for each treatment. Two weeks before harvest, fruits on each branch were sprayed with an appropriate treatment suspension until runoff. Twenty-four hours later, all fruits, including the control treatment, were sprayed with *M. laxa* spore suspension (Appendix Table 3). In 2020, 10 further trees of cultivar Kordia at NIAB East Malling in the same open-air orchard as 2019 were used; nine branches were selected from each tree and each branch was randomly assigned to one of the nine treatments. There were two application timings relative to the pathogen inoculation – ‘preventative’ and ‘curative’. For the ‘preventative’timing, the treatments were applied 24 h before *M. laxa* was applied; for the ‘curative’ timing, the treatments were applied 24 h after *M. laxa* was applied to the fruit. To avoid contamination between treatments, the branch was placed into a clear polythene bag when fruit were appleid with either biocontrol or pathogen suspension. Two weeks before harvest, fruits on each branch were sprayed with the ‘preventative’ treatments (two biological controls, fungicide and water as a control) until runoff. Twenty-four hours later, all fruits, excluding the control with no pathogen inoculum (‘no’), were sprayed with *M. laxa* spore suspension. Twenty four hours after *M. laxa* inoculation, the treatments were applied to the fruit on those branches allocated to the ‘curative’ timing (Appendix Table 4).

Two weeks after the ‘preventative’ timing treatments, all visibly healthy ripe fruits were harvested and stored on sterile trays: in 2019 at 20 °C and in 2020 at 4 °C. Throughout the incubation period, fruit with visible rot was recorded on days 1, 2 and 4 and removed immediately after assessment to prevent secondary contact spread. The assessment was carried out for 4 days in 2019 at which time the control treatment reached 100% rot, and until day 15 in 2020, generally considered the maximum shelf life (Habib et al., 2017). For each branch, data consisted of a total number of healthy fruit and rotted fruit at a given assessment time. Individual trees functioned as blocks. The percent fruit rot of each branch was logit transformed and then ANOVA was conducted to assess the treatment effects on the incidence of post-harvest fruit rotting. Individual contrasts (Appendix Table 1) were used to answer specific questions.

The climatic conditions in the field were typical of the season and similar across both years. In 2019, daily average temperature ranged from 12 °C to 26 °C; it was mostly sunny with light rain on the 7th of July, three days after inoculation. In 2020, daily average temperature ranged from 12 °C to 25 °C; it was mostly sunny with light showers on the 27th and 25th of July, the second week after inoculation.

Treatment with BCAs reduced the incidence of post-harvest fruit rotting in both years (Fig. 1). In 2019, application of either of the two biocontrol strains (B91 and Y126) two weeks before harvest, the day before inoculation, reduced the incidence of post-harvest fruit rot by 70–75% on day 1 and by 25–30% by day 4 (both P < 0.001) when incubated under ambient conditions (ca. 20 °C). There was little difference between the two biocontrol treatments.

**Fig. 1:**
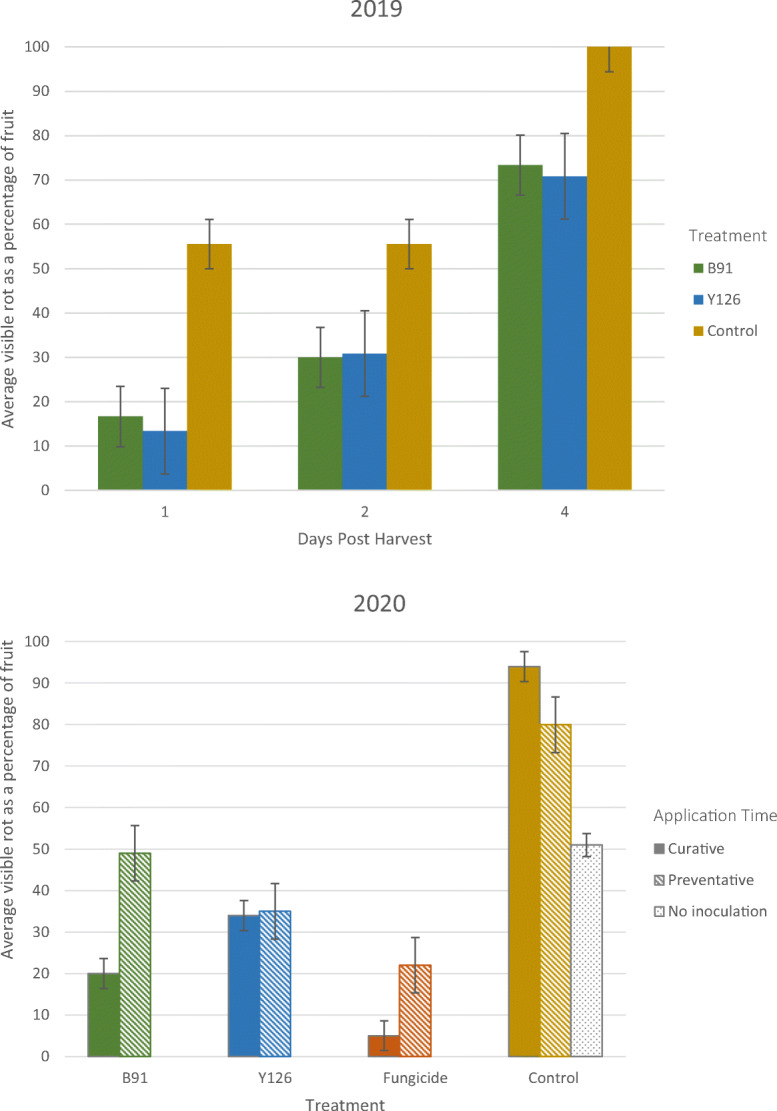
Percentage of cherry fruits with visible rot post-harvest in 2019 and 2020. Error bars show the standard error that was calculated using sqrt(p(1-p)/n) where n is the number total number of fruits per category and p is the proportion infected. For 2019, data are shown for all three assessments individually: days 1, 2 and 4 post-harvest. 90 fruits were assessed for each treatment (30 fruits per block). For 2020; the data shown are the overall percentages of cherry fruits with visible rot by day 15 of post-harvest storage at 4 °C. For each treatment and application time there were 100 fruits (10 per block). In 2019, three products (B91, Y126 and sterile distilled water [control] were applied 24 h before inoculation of fruit with *M. laxa*. In 2020, four products (B91, Y126, fungicide [Lunar sensation] and sterile distilled water [control]) were applied 24 h before (Preventative) or after (Curative) inoculation of fruit with *M. laxa*; the ‘No Inoculation’ control received only sterile distilled water and was not inoculated. In both years, fruits were inoculated with *M. laxa* two weeks before harvest

In 2020, an extra control treatment without inoculation of both *M. laxa* and biocontrol strains was added to assess the background levels of infection. Figure 1 shows that the background level of disease was very high, close to 50%. Nonetheless, the effects of biocontrol treatments were similar to 2019, with both the biocontrol microbes substantially reducing the incidence of post-harvest rot by 59% and 36% for Y126 and B91, respectively, for the ‘preventative’ application time, and 62% and 80% for the ‘curative’ application (Fig. 1; Appendix Table 2 contrast (a): P < 0.001). The fungicide performed better than the two BCAs (Appendix Table 2 contrast (c): P < 0.001).

Within the water control treatments, there was no significant difference in incidence due to application time (Appendix Table 2 contrast (b): P = 0.08). There was also no significant difference in incidence due to application time of the fungicide treatment (Appendix Table 2 contrast (d): P = 0.2), though the reduction was consistently slightly greater when fungicide was applied after the *M. laxa* inoculation (Fig. 1). Averaged over both spray timings, there was little difference in the post-harvest rots between Y126 and B91 (Appendix Table 2 contrast (e): P = 0.9). However, for B91 the application time had a strong effect: B91 was more effective when applied after the *M. laxa* inoculation (Appendix Table 2 contrast (f): P < 0.001). Unlike the fungicide and B91 treatments, there was little difference in the incidences of post-harvest rot between the two application times of Y126 (Appendix Table 2 contrast (g): P = 0.7).

The fungicide, as expected, reduced *M. laxa* infections and hence post-harvest rots. It was slightly more effective when it was applied after the *M. laxa* inoculation, despite its mode of action. The active ingredient fluopyram prevents spore germination of *M. laxa* so, to be effective, needs to be present on the fruit surface when spores arrive. The application before pathogen inoculation may have resulted in a lower concentration of fungicide on the fruit surface to interact with *M. laxa,* compared to the application after pathogen inoculation. In addition, for the ‘preventative’ application, a side-target effect of the fungicide on resident microbiomes may have led to the pathogen experiencing less competition from them (Busby et al., 2016).

B91 had a similar pattern to the fungicide, being slightly more effective when applied after the *M. laxa* inoculation. One important mode of action for B91 is through the production of toxins (Rungjindamai et al., 2013). Thus, we expected that B91 would work better when applied before *M. laxa* as it would enable the biocontrol to establish on the fruit surface and produce these toxins. The opposite was, however, observed. Because B91 was grown in liquid media for 24 h before application, it is possible that the toxins were present in high concentrations in the application solution. Another possibility could be a rapid reduction of the B91 population after its application. This should be considered when formulating the biocontrol microbe into a product. Indeed, ensuring high concentrations of these toxins within their product is the formulation strategy used for Serenade, a commercially formulated product of a specific *B. subtilis* strain (Yánez-Mendizábal et al., 2012). There was little difference between the treatments with fungicide (applied before inoculum) and B91 (applied after inoculum) showing the importance of the application time for biocontrol efficacy.

With Y126 there was no significant difference between the incidences of post-harvest fruit rot at the two inoculation times. Y126 works primarily through competition with the pathogen (Rungjindamai et al., 2013) so this observation demonstrates that it can compete successfully with *M. laxa* even when applied 24 h after *M. laxa* inoculation. However, biocontrol outcomes could be different if infection of fruit is via wounds: infection of wounded fruit is expected to be much quicker than infection of healthy and intact fruit; thus early establishment of a biocontrol population is expected to be critically important.

There is a need to investigate how the two biocontrol strains interact with other management strategies used to combat brown rot and how biocontrol could be successfully integrated with other pest and disease management practices within orchards. The poor survival of biocontrol microbes in the field has often been cited as a barrier to the effective deployment of BCAs in commercial agriculture and horticulture. However, applications closer to harvest have shown more promise (Sharma et al., 2009), as in the experiments reported here. Knowledge of the survival dynamics of biocontrol microbes in the field could assist in timing their applications more effectively. The difference in the incidence of post-harvest rot due to the timing of application relative to pathogen inoculation also shows the importance of understanding the ecology of the biocontrol microbes and their mode of action to optimise their efficacy.

Overall, these results suggest that both Y126 and B91 remain effective at 4 °C and that B91 applied after pathogen inoculation achieved an efficacy almost as good as the fungicide control. This is a promising step forward for the biological control of cherry brown rot, which may reduce reliance on fungicides in commercial orchards.

## Supplementary Information

ESM 1 (DOCX 22 kb)

## Data Availability

Not Applicable.
