# Supplementary material for: Field application of Bacillus subtilis and Aureobasidium pullulans to reduce Monilinia laxa post-harvest rot on cherry
Source: Eur J Plant Pathol. 2022 Apr 26;163(3):761–6. doi: 10.1007/s10658-022-02508-8 (PMC9213261; doi:10.1007/s10658-022-02508-8)
Supplement: Supplementary file 1 — (DOCX 22 kb) [file 10658_2022_2508_MOESM1_ESM.docx]

**Supplementary Information (SI)**

Article title: Field application of *Bacillus subtilis* and *Aureobasidium pullulans* to reduce *Monilinia laxa* post-harvest rot on cherry

Journal name: EJPP

Authors: Sophia Bellamy^1,2^, Michael Shaw^2^, Xiangming Xu^1^

Affiliations:

^1^NIAB EMR (Pest & Pathogen Ecology), East Malling, (Kent), UK

^2^University of Reading institution, (School of Agriculture, Policy and Development), Reading, (Berkshire), UK

Email: [sophia.bellamy@niab.com](mailto:sophia.bellamy@niab.com), [Xiangming.Xu@niab.com](mailto:Xiangming.Xu@niab.com), [m.w.shaw@reading.ac.uk](mailto:m.w.shaw@reading.ac.uk)

**Appendix Table 1:** Shows the individual contrasts used in the analysis of the 2020 data and the questions that they address.

| Treatment | Time | (a) Is there any control present? | (b) The trend over time, in the control? | (c) Is fungicide better or worse than BCAs? | (d) Does timing affect fungicide? | (e) Do BCAs differ? | (f) Is B91 better applied early? | (g) Is Y126 better applied early? |
| --- | --- | --- | --- | --- | --- | --- | --- | --- |
| B91 | After | 1 | 0 | 1 | 0 | 1 | 1 | 0 |
|  | Before | 1 | 0 | 1 | 0 | 1 | -1 | 0 |
| Y126 | After | 1 | 0 | 1 | 0 | -1 | 0 | -1 |
|  | Before | 1 | 0 | 1 | 0 | -1 | 0 | 1 |
| Fungicide | After | 1 | 0 | -2 | 1 | 0 | 0 | 0 |
|  | Before | 1 | 0 | -2 | -1 | 0 | 0 | 0 |
| SDW (control) | After | -3 | 1 | 0 | 0 | 0 | 0 | 0 |
|  | Before | -3 | -1 | 0 | 0 | 0 | 0 | 0 |

**Appendix Table 2:** Anova table for individual contrasts showing P-values stated in the paper. Results were generated in R using logit transformed data.

|  | Estimate | Std. Error | t value | Pr (>\|t\|) |
| --- | --- | --- | --- | --- |
| (Intercept) | -0.209 | 0.1003 | -2.09 | 0.04 |
| (a) Is there any control present? | -0.715 | 0.058 | -12.34 | < 0.001 |
| (b) The trend over time, in the control? | 0.355 | 0.201 | 1.77 | 0.08 |
| (c) Is fungicide better or worse than BCAs? | 0.292 | 0.082 | 3.56 | <0.001 |
| (d) Does timing affect fungicide? | -0.261 | 0.201 | -1.30 | 0.20 |
| (e) Do BCAs differ? | 0.021 | 0.142 | 0.15 | 0.88 |
| (f) Is B91 better applied early? | -0.730 | 0.201 | -3.64 | <0.001 |
| (g) Is Y126 better applied early? | -0.074 | 0.201 | -0.37 | 0.72 |

**Appendix Table 3:** Application times for the treatments and *M. laxa* for 2019.

| Treatment | Date M. laxa Applied | Date Treatment Applied | Harvested |
| --- | --- | --- | --- |
| B91 | 05/07/2019 | 04/07/2019 | 19/07/2019 |
| Y126 | 05/07/2019 | 04/07/2019 | 19/07/2019 |
| Fungicide | 05/07/2019 | 04/07/2019 | 19/07/2019 |
| Control | 05/07/2019 | 04/07/2019 | 19/07/2019 |

**Appendix Table 4:** Application times for the different treatments , application times and *M. laxa* for 2020.

| Treatment | Application | Date *M. laxa* Applied | Date Treatment Applied | Harvested |
| --- | --- | --- | --- | --- |
| B91 | Before | 16/07/2020 | 15/07/2020 | 30/07/2020 |
| Y126 | Before | 16/07/2020 | 15/07/2020 | 30/07/2020 |
| Fungicide | Before | 16/07/2020 | 15/07/2020 | 30/07/2020 |
| Control | Before | 16/07/2020 | 15/07/2020 | 30/07/2020 |
| B91 | After | 16/07/2020 | 17/07/2020 | 30/07/2020 |
| Y126 | After | 16/07/2020 | 17/07/2020 | 30/07/2020 |
| Fungicide | After | 16/07/2020 | 17/07/2020 | 30/07/2020 |
| Control | After | 16/07/2020 | 17/07/2020 | 30/07/2020 |
| Control (No) | NA | NA | 17/07/2020 | 30/07/2020 |
